# Improving reference prioritisation with PICO recognition

**DOI:** 10.1186/s12911-019-0992-8

**Published:** 2019-12-05

**Authors:** Austin J. Brockmeier, Meizhi Ju, Piotr Przybyła, Sophia Ananiadou

**Affiliations:** 1 National Centre of Text Mining, School of Computer Science, University of Manchester, Princess Street, Manchester, M1 7DN UK; 2 University of Delaware, 139 The Green, Newark, Delaware, 19716 USA; 3 Linguistic Engineering Group, Institute of Computer Science, Polish Academy of Sciences, Jana Kazimierza 5, Warszawa, 01-248 Poland; 4 The Alan Turing Institute, 96 Euston Road, London, NW1 2DB UK

**Keywords:** Active learning, Evidence-based medicine, Logistic regression, Machine learning, Text mining, Systematic review

## Abstract

**Background:**

Machine learning can assist with multiple tasks during systematic reviews to facilitate the rapid retrieval of relevant references during screening and to identify and extract information relevant to the study characteristics, which include the PICO elements of patient/population, intervention, comparator, and outcomes. The latter requires techniques for identifying and categorising fragments of text, known as named entity recognition.

**Methods:**

A publicly available corpus of PICO annotations on biomedical abstracts is used to train a named entity recognition model, which is implemented as a recurrent neural network. This model is then applied to a separate collection of abstracts for references from systematic reviews within biomedical and health domains. The occurrences of words tagged in the context of specific PICO contexts are used as additional features for a relevancy classification model. Simulations of the machine learning-assisted screening are used to evaluate the work saved by the relevancy model with and without the PICO features. Chi-squared and statistical significance of positive predicted values are used to identify words that are more indicative of relevancy within PICO contexts.

**Results:**

Inclusion of PICO features improves the performance metric on 15 of the 20 collections, with substantial gains on certain systematic reviews. Examples of words whose PICO context are more precise can explain this increase.

**Conclusions:**

Words within PICO tagged segments in abstracts are predictive features for determining inclusion. Combining PICO annotation model into the relevancy classification pipeline is a promising approach. The annotations may be useful on their own to aid users in pinpointing necessary information for data extraction, or to facilitate semantic search.

## Background

Evidence-based research seeks to answer a well-posed, falsifiable question using existing results and a systematic and transparent methodology. The evidence—for example, results of clinical trials—should be collected and evaluated without bias using consistent criteria for inclusion [1]. For certain cases [2], a research question can be decomposed into its PICO elements: patient/population, the intervention, comparator, and outcomes [3, 4]. Along with other aspects, such as study design, PICO elements are useful for formulating search queries for literature database searches [5] and mentions of PICO elements are key to screening the search results for relevance.

A standard approach for systematic reviews (and other review types such as rapid reviews [6] and scoping reviews [7]) is to perform screening initially using only the title and abstracts of a reference collection before obtaining and analysing a subset of full-text articles [1]. While faster and more cost effective than full-text screening, manually screening all reference abstracts is a protracted process for large collections [8], especially those with low specificity [9].

Technology-assisted reviewing seeks to foreshorten this process by only screening the subset of the collection most likely to be relevant [10–13]. This subset is automatically selected using information from a manual screening decisions either on another, ideally smaller, subset of the collection [14] or through multiple rounds of iterative feedback between a machine learning (ML) model and the human reviewer [15]. In effect, the machine ‘reads’ the title and abstract and scores the relevancy of the reference based on a model trained on relevant and irrelevant examples from the human reviewer. While previous studies [7, 16, 17] have shown the potential for time-savings, the underlying models treat each word equally and do not explicitly distinguish PICO elements within an abstract. As PICO elements are crucial for a human reviewer to making inclusion decisions or design screening filters [18], we hypothesise that a ML model with information on each reference’s PICO would outperform a similar model lacking this information.

**Fig. 1:**
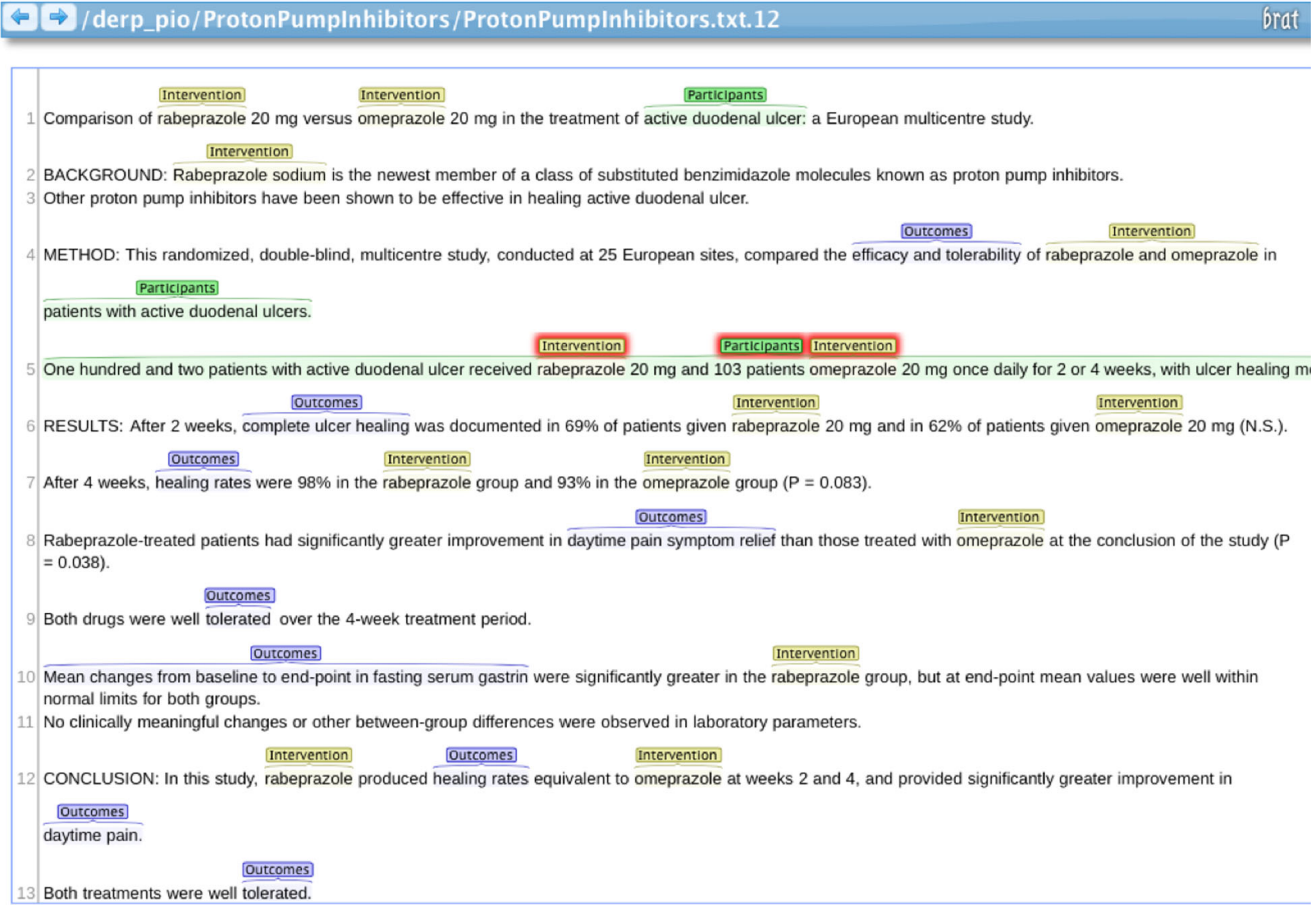
PICO recognition example. Visualisation of the trained model’s predictions of PICO elements within a reference (title and abstract) from the Proton Pump Inhibitors review. The interventions tags correspond to drug names, participant spans cover characteristics of the population, but erroneously include details of the intervention. The latter demonstrates the model’s ability to nest shorter spans within longer pans. The outcomes cover spans for qualitative and quantitative measures. Screenshot from the brat system [23]

**Fig. 2:**
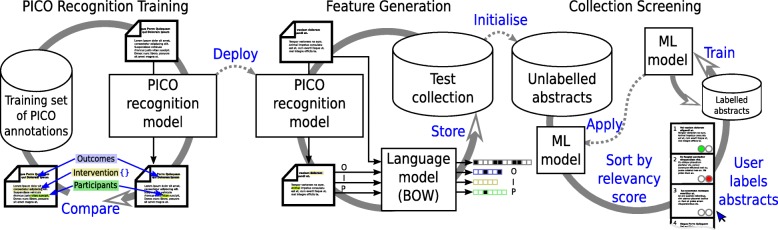
PICO recognition and abstract screening process. In the first phase, the PICO recognition model is trained to predict the PICO mention spans on a human annotated corpus of abstracts. In the second phase, a collection of abstracts is processed by the PICO recognition model and the results along with the original abstract are used to create a vector representation of each abstract. In the final phase, a user labels abstracts as being included (relevant) or excluded, these decisions are used to train a machine learning (ML) model that uses the vector representation. The ML model is applied to the remaining unlabelled abstracts, which are then sorted by their predicted relevancy, the user sees the top ranked abstracts, labels them, and this process repeats

Towards this aim, we propose a PICO recognition model that is able to automatically identify text describing PICO elements within titles and abstracts. The text fragments (contiguous sequences of words) are automatically identified using a named entity recognition model [19] trained on a manually annotated corpus of clinical randomised trial abstracts [20]. Underlying the success of the network is a vector representation of words that is pre-trained on a corpus of PubMed abstracts and articles [21]. The recognition model is based on a neural network architecture [22] that is enhanced to allow the extraction of nested spans, allowing text for one element to be contained within another element. For example, consider the sentence, $\footnotesize \underbrace {\text {Steroids}}_{intervention}\text { in }\underbrace {\underbrace {\text {paediatric}}_{population}~\underbrace {\text {kidney transplant}}_{intervention}\text {recipients}}_{population}\\\text { resulted in reduced }\underbrace {\text {acute rejection}}_{outcome}.$ The model’s predictions are illustrated in Fig. 1. The words in each of the PICO spans are correspondingly marked and treated as additional binary features (in a bag-of-words representation) for a ML model based on a previously validated model [17]. Figure 2 summarizes the whole process as a flowchart.

The performance of the abstract-level screening is evaluated on a standard data set collection of drug effectiveness systematic reviews [14, 24] (DERP I) by the Pacific Northwest Evidence-based Practice Center [25]. The results indicate consistent improvement using PICO information. Furthermore, we perform statistical analysis to identify words that when marked as belonging to a particular PICO element are significant predictors of relevancy and are more precise (higher positive predictive value) than the same words not constrained to the context of PICO mentions. This illustrates how automatically extracting information, obtained by a model trained on expert PICO annotations, can enrich the information available to the machine assisted reference screening.

### Related work

Previous work has shown that there are multiple avenues for automation within systematic reviews [26–28]. Examples include retrieval of high-quality articles [29–32], risk-of-bias assessment [33–36], and identification of randomised control trials [37, 38]. Matching the focus of the work, we review previous work on data extraction [39] to automatically isolate PICO and other study characteristics, can be methods for aiding abstract-level screening. The two are clearly related, since inclusion and exclusion criteria can be decomposed into requirements for PICO and study characteristics to facilitate search [40].

Extracting PICO elements (or information in broader schema [41]) at the phrase level [42–44] is a difficult problem due to the disagreement between human experts on the exact words constituting a PICO mention [45, 46]. Thus, many approaches [39] firstly determine the sentences relevant to the different PICO elements, using either rules (formulated as regular expressions) or ML models [42, 46–52]. Finer-grained data extraction can then be applied to the identified sentences to extract the words or phrases for demographic information (age, sex, ethnicity, etc.) [42, 48, 52–54], specific intervention arms [55], or the number of trial participants [56]. Instead of classifying each sentence independently, the structured form of abstracts can be exploited by identifying PICO sentences simultaneously with rhetorical types (aim, method, results, and conclusions) in the abstract [57–60]. More broadly, PICO and other information can be extracted directly from full text articles [61–65].

Rather than extract specific text, Singh et al. predict which medical concepts in the unified medical language system (UMLS) [66] are described in the full-text for each PICO element [67]. They use a neural network model that exploits embeddings of UMLS concepts in addition to word embeddings. The predicted concepts could be used as alternative features rather than just the extracted text. This would supplement manually added metadata such as Medical Subject Headings (MeSH) curated by the U.S. National Library of Medicine [68], which are not always available or have the necessary categorisations.

Our proposed approach differs from existing by both operating at the subsentence level (words and phrases) and using a neural network model for processing text [69] without hand-engineered features. In particular, the proposed approach uses an existing model architecture [19] originally designed for named entity recognition [70] to identify mentions of biomedical concepts such as diseases, drugs, anatomical parts [71, 72]. The model builds from previous neural architectures [22, 73, 74]. The model is jointly trained to predict population, intervention, and outcomes in each sentence in the abstract, and can handle nested mentions where one element’s mention (like an intervention) can be contained within another like a population. This capability is novel to this work, and in theory, can provide higher recall than methods that do not allow nested PICO elements.

Automatically identified PICO information can improve other automation tasks such as clinical question answering [51] and predicting clinical trial eligibility [75, 76]. Likewise, inclusion and exclusion criteria can be decomposed into requirements for PICO and study characteristics to facilitate search [40]. Recently, Tsafnat et al. have shown the screening ability of automatic PICO extraction [18] for systematic reviews. They use manually designed filters (using dictionaries and rules) [77, 78] for key inclusion criterion, mentions of specific outcomes, population characteristics, and interventions (exposures) to filter collections with impressive gains. Our goal is to replace the manually designed filters with ML modelling that leverages the automatically extracted PICO text to determine an efficient filter. A variety of ML models (different classifiers, algorithms, and feature sets) have been proposed for screening references for systematic reviews [14, 15, 79–95]. Yet, to our knowledge none of relevancy classifiers have used as input the output of PICO recognition.

## Methods

The machine learning methodology consists of two main blocks: PICO recognition and relevancy classification. The two steps share some common text pre-processing. To pre-process the text in titles and abstracts, sentence boundaries are determined using the GENIA sentence splitter^1^ [96], which was trained on the GENIA corpus [97, 98]^2^. Within each sentence, GENIA tagger^3^ is used to determine the boundaries between words and other tokens and also the lemmata (base form) of each word [99]. Capitalisation is ignored and lowercase is used for words and lemmata. Additionally, for the PICO recognition each digit is mapped to a zero [69].

### PICO recognition model

The PICO annotations have the hierarchical categorisation given in Table 1 where the top-level categories consist of population, intervention/comparator, and outcomes—the comparators are merged into interventions [20]. The annotation is performed in two passes: firstly, top-level spans are identified, and secondly, spans within these are further annotated with the fine-grained types. In this manner, spans corresponding to the fine-grained types are nested within typically longer spans with top-level PICO types.

**Table 1:** The top-level and fine-grained PICO elements in the training set for the PICO recognition model

Top-level	Patient-population-problem	Intervention/Comparator	Outcome
Fine-grained	Age	Control	Adverse effect
	Condition	Educational	Mental
	Sample size	Pharmacological	Mortality
	Sex	Physical	Pain
		Psychological	Physical
		Surgical	Other
		Other	

Following this annotation, the recognition model is trained to firstly extract fine-grained entities, which are under the top-level PICO. Then it extracts the spans corresponding to the top-level PICO elements. To achieve this, the training data consists of an ordered list of IOB tagging [100] sequences for each sentence that mark the beginning (B) and inside (I) of each span, as well as tokens outside (O) of these spans. The lists begin with fine-grained shorter spans and move to top-level longer spans.

As described in detail [22], the network architecture for the recognition model consists of three main layers: an embedding layer, a sequence processing layer, and a output layer. Firstly, the embedding layer takes as input the sequence of tokens and the character sequence within each token and outputs a vector representation. Each token is represented using the concatenation of word embeddings [101] and representations based on processing character embeddings [102] with a bidirectional long short-term memory network (biLSTM) [103] that employ a forward and reverse LSTM [104] and concatenate the output. Words that are not found in the pre-trained word embeddings are mapped to a common vector, which is further trained by randomly dropping words (50% chance) that occur only once in the training corpus. The second layer processes the sequence of representations using another biLSTM. The third layer is an affine projection of this representation to produce the unitary potential for each of the possible tags in a conditional random field (CRF) model [105], which also models the transition probabilities between tags. Due to the IOB tagging scheme, there are 2×(3+17)+1=41 tags corresponding to beginning or inside of one of the 20 possible PICO categories (3 top-level and the 17 fine-grained) and the outside tag. The Viterbi algorithm [106] is used to efficiently infer the most likely sequence of tags marking the spans.

To make predictions of nested spans, the second layer and third layers are iteratively applied to the output of the second layer from the previous iteration until there are no more predicted spans. Specific dimensions of network architecture are detailed in Table 2. Other choices were not explored.

**Table 2:** Details of the 3-layer network architecture for the PICO recognition model

Layer		Size	Source
1a	Word embedding	200	[21], not updated
1b	Character embedding	28	trained from random initialisation
1c	Character-based word representation	2 ×28	biLSTM applied to 1b
1d	Combined embedding	256	concatenation of 1a and 1c
2	Recurrent layer	2 ×128	biLSTM over 1d
3	Linear layer	41	affine projection of 2
	CRF output	1	most likely sequence of tags

The network parameters are adjusted to maximise the log likelihood of training sentences for the CRF [69]. Stochastic first-order optimisation is performed using batches of sentences, gradient clipping, and Adam [107]. Dropout [108], weight decay (*L*_2_-regularisation), and early stopping are employed to prevent overfitting. Hyper-parameters are selected using Bayesian optimisation [109], using the design described in [19], on a development portion of the training set with the F1-score of the span-level predictions as the metric.

### Relevancy classification model

The relevancy classifier is trained on screening decisions (represented as binary variables indicating inclusion or exclusion). The predictions of the classifier on the unseen references are used to prioritize them, presenting those that are most likely to be relevant. The text processing and feature set follows the description of RobotAnalyst [17], a web-based system that uses ML to prioritise relevant references. The feature set consists of a bag-of-words (BOW) representation of the title, another BOW for the title and abstract combined, and the topic distribution of the title and abstract text.

Topic distributions for title and abstract text are inferred from an LDA topic model [110] with *k*=300 topics using MALLET [111]. The text is filtered to words consisting of alphabetic characters with initial or internal punctuation that are not on the stop word list. Topic model hyperparameters are initialized as *α*=1/*k* and *β*=1/100 with optimisation every 50 iterations. The topic proportions for each reference are normalised using the *L*_2_ norm.

For the baseline model, the two contexts are title or combined title and abstract. The BOWs are formed from lemmata (base forms) of the occurring words. Included lemmata consist of more than one character, have at least one letter or number, and are not found in a list of stop words^4^. The BOW is a sparse binary vector representing whether or not a word occurred in the given context. Each BOW is normalised to have a Euclidean (*L*_2_) norm of 1 for each reference, except when the bag is empty.

An additional feature set from the PICO recognition consists of a BOW for each of the three course-grained element types patient, intervention, and outcome (comparator is considered an intervention) recognised within the title or abstract. Although finer-grained spans are also annotated and recognised by the model, they were mapped back to the basic PICO types after recognition. In summary, the proposed model uses 5 BOWs. Note that these representations are not disjoint, as a word occurring within a PICO span would both be counted in the general BOW and in the corresponding PICO category BOW.

The classifier is a linear model implemented in LIBLINEAR [112]. While RobotAnalyst uses a support vector classifier, we adopt a logistic regression model with *L*_2_-regularisation.^5^ The amount of regularisation is controlled by the constraint violation cost parameter *C*, which is fixed at *C*=1.

### Identifying words with PICO-specific relevancy

We perform two statistical tests to identify words that are both predictive of relevancy for a particular PICO context, and are more predictive than occurrences of the word when it is not restricted to be within the context of a PICO mention. Firstly, for each context category, we compute each word’s correlation with relevancy labels using Pearson’s *χ*^2^ test statistic for independence. Secondly, for each context-word pair, we compute the positive predictive value (the ratio of the number of included documents containing the word to the total number of documents containing the word) and use Leisenring et al.’s generalised score statistic for equality of positive predictive value [113, 114] to see if the PICO-specific occurrence is significantly more predictive than the word’s unrestricted occurrence. The set of PICO-predictive words are those with a significant *χ*^2^ statistic and a positive predictive value both higher and significantly different than the unrestricted context, using a significance level of 0.01 for both tests.

### Datasets and simulation

A corpus of annotated references [20, 115] is used for training and evaluation the PICO recognition model. The corpus consists of 4,993 references, a subset of 4,512 are used for training and development (4,061/451). The remainder contains 191 for testing the coarse-grained spans. The remainder also contains 96 that were not used for training since they lacked at least one of the PICO elements, and 194 references which are part of a set of 200 assigned for testing fine-grained labelling. After sentence splitting, there are 43,295 and 4,819 sentences in the training and development sets, respectively.

The DERP collections [24, 116] are used to test whether including the PICO features will improve the prioritisation of relevant references using simulated screening. Table 3 describes the collections for the different reviews.

**Table 3:** DERP systematic review descriptive statistics

Review	Inc.	Exc.	Tot.	Prev.
ACE Inhibitors	2544	41	2503	1.61%
ADHD	851	20	831	2.35%
Antihistamines	310	16	294	5.16%
Atypical Antipsychotics	1120	146	974	13.04%
Beta Blockers	2072	42	2030	2.03%
Calcium Channel Blockers	1218	100	1118	8.21%
Estrogens	368	80	288	21.74%
NSAIDS	393	41	352	10.43%
Opioids	1915	15	1900	0.78%
Oral Hypoglycemics	503	136	367	27.04%
Proton Pump Inhibitors	1333	51	1282	3.83%
Skeletal Muscle Relaxants	1643	9	1634	0.55%
Statins	3465	85	3380	2.45%
Triptans	671	24	647	3.58%
Urinary Incontinence	327	40	287	12.23%

Abbreviated columns correspond to the number of inclusions (relevant references), exclusions, total number of references, and the prevalence (percentage of inclusions compared to total)

The simulation is modelled after the RobotAnalyst framework [17], where the classification model is updated at multiple stages during the screening process. Specifically, we run 100 Monte Carlo simulations. In each simulation, we begin with a random batch of 25 references. If this batch contains any relevant references, this forms the initial training set, otherwise batches of 25 are sampled randomly and appended to the training set until at least one relevant reference is found. Given the training set, a classifier is trained and applied to the remaining references. The references are prioritised by the classifier’s score, which is proportional to the posterior probability of being relevant (using a logistic regression model). The 25 highest ranked references are then included in the training set, a classifier is retrained, and so on. This continues until all references are screened. This iterative process is readily comparable to relevance feedback methods [117].

To compare against other baselines from the literature we also use a stratified 2-fold setting, where half of the inclusions and half of the exclusions are used for training. Internal results are reported for the average of 100 Monte Carlo trials of stratified training with 50% of each class for training and 50% for testing.

To test the wider applicability of the methodology we applied it to five additional collections introduced by Howard et al. [95]. Four of the collections were produced by the National Institute of Environmental Health Sciences’s National Toxicology Program’s Office of Health Assessment and Translation (OHAT), and the fifth was produced by the Edinburgh CAMARADES group [118]. Table 4 describes the collections for the different reviews.

**Table 4:** OHAT and COMARADES systematic review descriptive statistics

Review	Inc.	Exc.	Tot.	Prev.
PFOA/PFOS and Immunotoxicity	6331	95	6236	1.50%
Bisphenol A (BPA) and Obesity	7700	111	7589	1.44%
Transgenerational Inheritance of Health Effects	48638	765	47873	1.57%
Fluoride and Neurotoxicity in Animal Models	4479	51	4428	1.14%
Neuropathic Pain	29207	5011	24196	17.16%

Abbreviated columns correspond to the number of inclusions (relevant references), exclusions, total number of references, and the prevalence (percentage of inclusions compared to total)

### Evaluation

Firstly, the PICO recognition model is evaluated by its ability to identify top-level (patient, intervention, and outcome) mentions as annotated by experts. Performance is calculated in terms of the model’s recall and precision at the level of individual tokens. Each token is treated as an individual test case. True positives for each category are tokens in the category’s span that matches the one assigned by the model, and false positives are tokens assigned to the category by the model but not in the original span. This solves the problem of comparing two spans that have matching category, but partially overlapping spans.

The performance is also calculated at the document level in terms of the set of included words. This is a looser evaluation that tests whether the annotated PICO words would be captured when each document is represented as filtered BOW with lemmata, which using the same processing (removing single letter tokens, stop words, etc.) as the BOW for the relevancy classification model. In other words, the document-level matching tests how well individual documents could be retrieved by searching for words within specific PICO contexts. The evaluation uses a held out test set from the same collection as the recognition model training data [20].

Secondly, we test the hypothesis that adding automatically recognised PICO elements to the feature set improves the prioritisation of relevant references. In this setting, the main objective is to prioritise references such that relevant references are presented as early as possible. To compare against baselines from the literature we use both a two-fold relevancy prioritisation [84, 95, 119], and a relevancy feedback setting [120, 121]. In both cases, references with the highest probability of being relevant are screened first [88, 89, 91, 94, 122], like in relevance feedback [117].

As an internal baseline for BOW we consider an average of context-dependent word vectors. Word vectors are trained using algorithms, such as word2vec [123] and GloVe [124], on large corpora such that the vector-space similarity among words reflects the words’ distributional similarity: words with similar vectors appear in similar contexts. In comparison, with BOW each word is assigned a vector orthogonal to the rest, such that all words are equally dissimilar. Word vectors perform well on a variety of language tasks, and even better performance is possible when the vector representation of a word depends on its surrounding context [125]. In this case, the context-dependent word vector is computed by the hidden layers of a neural network trained on language modeling tasks. As suggested by a reviewer, we use the context-dependent word vectors from the BERT language model [126], specifically the BioBert model trained on *PubMed* abstracts to better reflect the language of biomedical research papers [127]. For each PICO mention, we compute the average of the output vectors of the last layer hidden of the model for all tokens covered by the span, and then average these for a given PICO category. The BERT representation of abstracts is obtained in the same way, except we average across the vectors for all of the abstract’s tokens.

Following previous work, we quantify the performance in terms of work saved over sampling at 95% recall (*WSS*@95%) [14]. This is computed as the proportion of the collection that remains after screening 95% of the relevant reference and subtracting 5% to account for the proportion expected when screening in random order. The recall after screening *i* references is

1 $$\begin{array}{*{20}l} \textit{recall}(i)&=\frac{\textit{TP}(i)}{\textit{TP}(i)+\textit{FN}(i)}, \end{array} $$

where *TP*(*i*) is the number of relevant references found and *FN*(*i*) is the number of relevant references that have not been screened. Likewise, *FP*(*i*) denotes the number of irrelevant references found, and *TP*(*i*)+*FP*(*i*)=*i*. Let *i*_R95_ denote the number of references screened when 95% recall is firstly achieved. Precisely,

2 $$\begin{array}{*{20}l} i_{\textrm{R95}}&=\min_{\substack{ i \in \{1,\ldots,N\} \\ \textit{recall}(i)\geq 0.95}} i. \end{array} $$

Under random ordering the expected value for *i*_R95_ is 95*%N*, where *N* denotes the total number of references. Work saved is $\frac {N-i_{\textrm {R95}}}{N}$, and

3 $$\begin{array}{*{20}l} \notag \text{\textit{WSS}@95\%}&= \frac{N-i_{\textrm{R95}}}{N} - 5\% \\ &= 95\%-\frac{i_{\textrm{R95}}}{N}, \end{array} $$

where *N* denotes the total number of references. The metric is intended to express how much manual screening effort would be saved by a reviewer that would stop the process after finding 95% of the relevant documents. While this metric is useful to compare algorithms, in practice a reviewer will not be able to recognise when 95% recall has been obtained and thus the work saving is a theoretical one, unless a perfect stopping criterion is available.

## Results

The test set of 191 abstracts [20, 115] is used to evaluate the model’s PICO annotation. The token-wise performance for the three categories is reported in Table 5. The model achieves an F-1 score (geometric mean of precision and recall) of 0.70 for both participants and outcomes, and 0.56 for interventions. The latter is caused by a much lower recall of 0.47. The performance metrics are higher for document-level matching, which uses the same processing (lemmatisation, removing single letter tokens, stop words, etc.) as the BOW for the relevancy classification model. For outcomes, a promising recall of 0.81 is achieved.

**Table 5:** PICO recognition performance in terms of a token-wise evaluation and a document-level filtered bag-of-words (BOW)

	Token-wise			Document-level BOW		
	Precision	Recall	F-1	Precision	Recall	F-1
Participants	0.81	0.62	0.70	0.86	0.71	0.78
Interventions	0.69	0.47	0.56	0.83	0.52	0.64
Outcomes	0.66	0.75	0.70	0.73	0.81	0.77

The results of relevancy feedback experiment are in Table 6 with the column labelled LR corresponding to the baseline set of features from RobotAnalyst with logistic regression, and PICO indicating the model with the additional PICO bag-of-words features. On average, the inclusion of PICO features increases the work saved metric by 3.3%, with substantial gains for the Opioids and Triptans collections.

**Table 6:** Relevancy feedback performance in terms of *WSS*@95% on DERP systematic review collections

	[120]	[121]	LR	PICO	*Δ*
ACE Inhibitors	74.3	*82.7	74.7	74.4	-0.3
ADHD	67.9	*82.1	67.5	68.9	1.4
Antihistamines	*24.5	17.7	-1.7	-1.9	-0.1
Atypical Antipsychotics	18.0	*33.6	18.0	20.5	2.5
Beta Blockers	65.0	*68.5	54.7	55.7	1.1
Calcium Channel Blockers	17.3	12.8	*47.6	47.1	-0.5
Estrogens	22.6	28.5	36.6	*39.1	2.4
NSAIDS	*77.4	64.1	60.9	63.1	2.2
Opioids	9.0	17.4	19.5	*34.1	14.6
Oral Hypoglycemic	13.5	*15.9	6.9	9.2	2.3
Proton Pump Inhibitors	19.7	21.0	*21.2	18.3	-2.9
Skeletal Muscle Relaxants	*58.6	29.9	25.9	32.4	6.5
Statins	27.8	*43.7	42.9	43.3	0.3
Triptans	39.6	*54.1	34.3	52.4	18.1
Urinary Incontinence	20.8	41.6	44.8	*46.4	1.6
Average	37.1	40.9	36.9	40.2	3.3

^*Δ*^indicates the change between adding the PICO features to the baseline logistic regression classifier (LR)

^*^indicate best performance per review

We compare these results against two baselines that use relevancy feedback rather ML. The first baseline is a relevance feedback system exploiting the lexical network induced by shared word occurrence [120]. This is a strong baseline as it uses a deterministic seed for retrieval based on custom set of terms in the research questions and the search strategy (in particular the inclusion criterion) and proceeds with relevance feedback adding one reference at a time. Ji et al. follow the same experiment and for a fair comparison we report their results for the case when parameters are fixed (*DT*=1) across collections using SNOMED-CT and MeSH features for a semantic network [121]. The overall performance with the PICO features is comparable to the semantic network based relevance feedback [121]. This is encouraging since the latter uses a human selected seed query, versus the random initialisation for the proposed method.

Other baselines from the literature only report results in the stratified 2-fold setting. The first baseline [84] uses a naive Bayes classifier, and the reported values are the average across five 2-fold cross-validations, in each of the 10 runs the WSS value for a threshold with at least 95% recall is reported. This includes a weight engineering factor for different groups of features that is maximised on the training set. The second baseline is an SVM-based model [79, 119] with the feature set that performed the best consisting of abstract and title text, MeSH terms, and Meta-map phrases. The final baseline [95] uses cross-validation on the training sets to select the following hyperparameters: the number of topics, the regularisation parameter, and the inclusion or exclusion of additional bigram, trigram, or MeSH term features. The reported values are an average across 25 Monte Carlo trials.

The results are reported in Table 7. The inclusion of PICO features improves the work saved performance metric versus the default logistic regression model, with an average improvement of 1.6%. The results are competitive against the earlier baselines, but the cross-validation selection of hyperparameters [95] yields the best average performance. Searching for these hyperparameters using cross-validations is computational demanding, especially in the relevance feedback setting, where there is not a large initial training set, but rather a different training set at each stage.

**Table 7:** Two-fold relevancy prediction in terms of *WSS*@95% on DERP systematic review collections

	[84]	[119]	[95]	LR	PICO	*Δ*
ACE Inhibitors	52.3	73.3	*80.1	78.5	77.6	-0.9
ADHD	62.2	52.6	*79.3	75.5	74.5	-0.9
Antihistamines	14.9	*23.6	13.7	4.9	5.0	0.1
Atypical Antipsychotics	20.6	17.0	*25.1	19.9	20.9	1.0
Beta Blockers	36.7	46.5	42.8	*55.5	54.1	-1.4
Calcium Channel Blockers	23.4	43.0	*44.8	38.8	39.3	0.6
Estrogens	37.5	41.4	*47.1	41.0	43.7	2.7
NSAIDS	52.8	67.2	*73.0	65.3	66.5	1.2
Opioids	55.4	36.4	*82.6	53.3	57.0	3.7
Oral Hypoglycemic	8.5	*13.6	11.7	7.1	8.9	1.8
Proton Pump Inhibitors	22.9	32.8	*37.8	32.6	31.0	-1.6
Skeletal Muscle Relaxants	26.5	37.4	*55.6	40.1	45.3	5.3
Statins	31.5	*49.1	43.6	42.2	44.3	2.1
Triptans	27.4	34.6	41.2	40.6	*51.2	10.5
Urinary Incontinence	29.6	43.2	*53.0	52.4	52.4	0.0
Average	33.5	40.8	48.8	43.2	44.8	1.6

^*Δ*^indicates the change between adding the PICO features to the baseline logistic regression classifier (LR)

^*^indicate best performance per review

Results on the additional OHAT and CAMARADES collections are shown in Table 8. The inclusion of PICO features improves performance on three of the five collections, with an average improvement of 0.3%.

**Table 8:** Two-fold relevancy prediction in terms of *WSS*@95% on OHAT and CAMARADES systematic review collections

	[95]	LR	PICO	*Δ*
PFOA/PFOS and Immunotoxicity	80.5	84.0	*84.6	0.7
Bisphenol A (BPA) and Obesity	75.2	77.9	*78.6	0.8
Transgenerational Inheritance of Health Effects	71.4	*74.3	*74.3	0.0
Fluoride and Neurotoxicity in Animal Models	87.0	89.3	*89.4	0.1
Neuropathic Pain	*69.1	64.3	64.1	-0.1
Average	76.6	77.9	78.2	0.3

^*Δ*^indicates the change between adding the PICO features to the baseline logistic regression classifier (LR)

^*^indicate best performance per review

Considering all 20 collections, the addition of PICO features yields a significant improvement in two-fold *WSS*@95% performance over the baseline logistic regression classifier as assessed by a one-sided sign-test (p-value of 0.0207) at a significance level of 0.1.

**Fig. 3:**
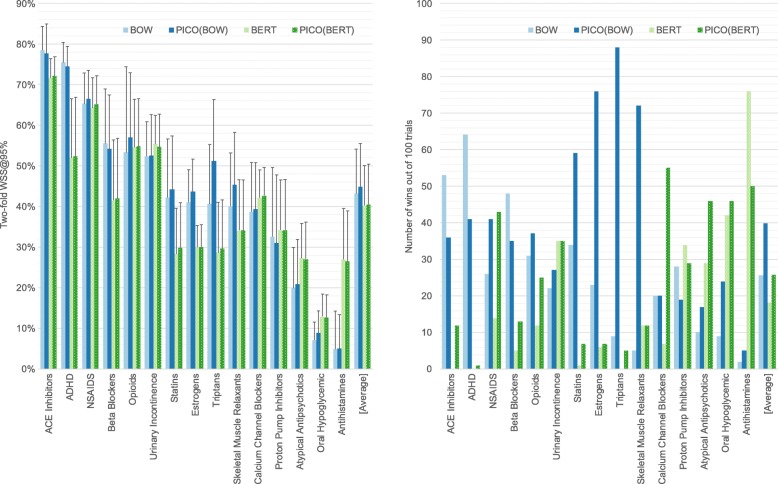
Comparison of BOW and BERT word vectors as the machine learning representation. The two-fold relevancy prediction performance is reported in terms of WSS@95% across the DERP collections, sorted by BOW performance. In each group, the different colored bars correspond to BOW, BOW including PICO features, BERT, and BERT including PICO features. Bar heights are the average across 100 Monte Carlo trials. In the WSS@95% plot, the upper error bars indicate the standard deviation across the 100 Monte Carlo trials

In Fig. 3, we report the two-fold performance on the DERP collections comparing BOW to BERT with and without the additional PICO features. On this internal comparison, we log and report the number of times a representation performs best across the Monte Carlo trials. BERT performs better on the most difficult collections, but on average, BOW outperforms BERT. Interestingly, the collections that have the highest gain between PICO(BOW) and BOW—Statins, Estrogens, Triptans, and Skeletal Muscle Relaxants—also have a large gap between BOW and BERT. This highlights the utility of the precision that BOW and PICO tagging provide. To assess whether the performance differences were statistically significance, we consider the performance rank of each representation per collection. The average ranks (where the best performing is assigned rank 1) are 2.1 for PICO(BOW), 2.4 for PICO(BERT), 2.7 for BOW, and 2.9 for BERT. The differences in average rank are not significant using a Friedman test at a significance level of 0.1.

To better illustrate the methodology, a subset of PICO features selected by the hypothesis tests for strong relevancy are shown in Tables 9 and 10. The two examples over the cases where the inclusion of PICO features lowered the performance on the Proton Pump Inhibitor review, and raised the performance on the Triptans review. In both cases, the strongly relevant features are clearly indicative of key inclusion aspects. For example, given an occurrence of the word ‘complete’ there is less than a 50% chance of the reference being relevant; however, within the spans marked as outcome the chance is over 70%. The lower performance in the case of the Proton Pump Inhibitor review corresponds to a lower positive predictive value on these features.

**Table 9:** PICO features with strong relevancy within the Proton Pump Inhibitors systematic review

PICO		PPV		TP/FP	
Tag	Lemma	PICO	BOW	PICO	BOW
O	relief	0.21	0.17	21/78	22/111
O	healing	0.13	0.11	33/215	33/264
O	heartburn	0.15	0.11	16/94	16/125
O	pain	0.15	0.12	14/79	14/98
P	oesophagitis	0.14	0.11	13/77	13/107
O	rate	0.07	0.07	35/439	35/501
P	grade	0.15	0.08	8/44	8/90
O	safety	0.10	0.08	11/94	11/122
P	reflux	0.07	0.05	23/311	23/441

Positive predictive value (PPV) is the proportion of true positives (TP) to the total number of TP and false positives (FP). Each TP corresponds to an inclusion containing the feature; each FP corresponds to an exclusion containing the feature

**Table 10:** PICO features with strong relevancy within the Triptans systematic review

PICO		PPV		TP/FP	
Tag	Lemma	PICO	BOW	PICO	BOW
O	relief	0.68	0.61	96/46	106/67
O	headache	0.53	0.43	130/113	161/212
P	migraine	0.50	0.41	138/138	198/281
P	treat	0.78	0.59	49/14	124/85
O	pain	0.59	0.52	90/63	96/89
O	severe	0.80	0.60	40/10	89/60
O	moderate	0.79	0.63	34/9	94/55
O	response	0.59	0.49	51/35	71/75
I	sumatriptan	0.43	0.41	141/187	145/211
O	mild	0.73	0.53	29/11	71/62
O	migraine	0.51	0.41	74/70	198/281
O	functional	0.81	0.56	21/5	25/20
O	effective	0.82	0.47	18/4	106/120
O	patient	0.67	0.43	26/13	194/253
O	complete	0.71	0.47	15/6	36/40
O	reduction	0.64	0.42	16/9	30/42
O	reduce	0.87	0.38	7/1	39/63
O	migraine-specific	0.80	0.50	8/2	10/10

Positive predictive value (PPV) is the proportion of true positives (TP) to the total number of TP and false positives (FP). Each TP corresponds to an inclusion containing the feature; each FP corresponds to an exclusion containing the feature

## Discussion

The results indicate that the additional PICO tagging is useful for improving machine learning performance in both the two-fold and relevancy feedback scenarios with a bag-of-words representation. This could only be the case if the additional features carry information about the relevancy decisions and are not redundant with the existing feature sets. These questions are answered by statistical analysis, which shows that when restricted to a specific PICO context certain words are more reliable predictors. As inclusion criteria are often stated in terms of PICO (and other study characteristics) this is not a surprising result, but nonetheless, requires a well-trained PICO recognition model to transfer the knowledge from the training set of annotations. In a way, the proposed methodology connects with previous work on generalisable classifiers that can learn from the screening decisions of other systematic reviews [128].

Furthermore, PICO tagging is an interpretable process meant to emulate human annotation and can readily be used by reviewers themselves. For instance, highlighting the mentions of outcomes may accelerate data extraction, since identifying outcome measures and data are a critical step in many systematic reviews. In the context of the ML model, the influence of a specific PICO feature in prioritising an abstract can be assessed by the corresponding coefficients of the logistic regression model. This can be used to check which of the PICO categories has contributed the most to the score assigned to a certain abstract—for example, the presence of an outcome-specific word with a relatively large coefficient. If this raises doubts, the text spans assigned to this type can be verified. The ability to interact with the model in such ways would increase its interpretability, which could aid a user in understanding and trusting the current model’s predictions [129]. While this can be done for all of the words, the semantics, sparsity and higher precision of PICO features make them more meaningful.

There are a number of avenues for future work. The first is to consider PICO tagging in new systematic reviews. The simulation results remains a surrogate for actual live screening evaluation as was performed by Przybyła et al. [17]. In practice, users may benefit from more precise queries where search terms are restricted to appear in PICO recognised spans, or integrated into additional facets for semantic search [130]. That is, the semantic classes of interventions and outcomes may be useful for users to search large collections and databases. For example, if instead of searching for a phrase or word describing an outcome measure in the whole text of the references, a reviewer would be able to search just within the fragments categorised as outcomes, the results would better align with the reviewer’s intention. The word ‘reduce’ in Table 10 is a strong example, where only 8 results with 7 being relevant are returned for ouctome-specific usage compared to 102 results with only 39 relevant in general. This demonstrates that a query-driven approach with PICO tagging has the potential to greatly reduce screening efforts needed to obtain an initial seed of relevant documents. User selected queries could be combined with RobotAnalyst’s ability to prioritise the results based on relevance predictions. Essentially, this would combine the approach proposed here with the ability for human design [18] of screening rules using PICO classes. Finally, in this work the fine-grained PICO recognition was not evaluated, but this may be useful to highlight population information (sample size, age, sex, condition).

During peer review, it was noted that the DERP collections also contain the reasons for most exclusions. Reasons for exclusions are often recorded in systematic reviews, and may be coded using PICO categories. Thus, a system with PICO-specific feature sets has the potential of incorporating the additional information into a ML model. This is an interesting area for future work.

Finally, we note that the proposed methodology is not able to beat relevancy screening baselines previously reported in the literature. This can largely be attributed to differences in evaluation. For the relevancy feedback experiments, the baseline methods [120, 121] start from deterministic queries that use expert knowledge of the inclusion criteria, versus the random initialisation for the propose method. In the case of two-fold predictions, the best performing method [95] uses cross validation to select the best from among different hyperparameters combinations, including distinct feature set choices. This would require additional computation in the online setting and it is not clear if this approach would perform well in the limited data setting (without access to half of the inclusions).

## Conclusion

Screening abstracts for systematic reviews requires users to read and evaluate abstracts to determine if the study characteristics match the inclusion criterion. A significant portion of these are described by PICO elements. In this study, words within PICO tagged segments automatically identified in abstracts are shown to be predictive features for determining inclusion. Combining PICO annotation model into the relevancy classification pipeline is a promising approach to expedite the screening process. Furthermore, annotations may be useful on their own to aid users in pinpointing necessary information for data extraction, or to facilitate semantic search.

## Data Availability

The datasets supporting the conclusions of this article are available in the Drug Effectiveness Review Project (DERP) repository [24], the EBM-NLP corpus [115], and as additional files [95].

## Footnotes

1 http://www.nactem.ac.uk/y-matsu/geniass/

2 The boundaries are based on punctuation and are unable to correctly split abstracts with lists of unterminated sentences.

3 http://www.nactem.ac.uk/GENIA/tagger/

4 http://members.unine.ch/jacques.savoy/clef/

5 Preliminary experiments showed logistic regression consistently improved the relevancy prioritisation.
